# Evaluation of candidemia in epidemiology and risk factors among cancer patients in a cancer center of China: an 8-year case-control study

**DOI:** 10.1186/s12879-017-2636-x

**Published:** 2017-08-03

**Authors:** Ding Li, Rui Xia, Qing Zhang, Changsen Bai, Zheng Li, Peng Zhang

**Affiliations:** 1 Department of Clinical Laboratory, Tianjin Medical University Cancer Institute and Hospital, National Clinical Research Center of Cancer, Key Laboratory of Cancer Prevention and Therapy, Tianjin, Huanhu West Road, Ti-Yuan-Bei, Hexi District, Tianjin, 300060 People’s Republic of China; 2 Intensive Care Unit, Tianjin Medical University Cancer Institute and Hospital, National Clinical Research Center of Cancer, Key Laboratory of Cancer Prevention and Therapy, Tianjin, Huanhu West Road, Ti-Yuan-Bei, Hexi District, Tianjin, 300060 People’s Republic of China

**Keywords:** Candidemia, *Candida*, Bloodstream infection, Cancer, Risk factor

## Abstract

**Background:**

Candidemia is the worldwide life-threaten disease, especially in cancer patients. This study was aimed to identify and evaluate the risk factors of candidemia in cancer patients, which will prompt the improvement on current therapeutic strategies and prognosis.

**Methods:**

A retrospective, case-control study was conducted from inpatients of Tianjin Medical University Cancer Institute and Hospital, during 2006 to 2013. Analyses were performed between cancer patients with candidemia as study case, and patients with bacterial bloodstream infections as control. Each case was matched up with two controls, for gender and inpatient duration. *Candida* species, clinical characteristics, risk factors and outcomes were reviewed in details.

**Results:**

Total number of 80 cases and 160 controls were enrolled and analyzed in this study. *Candida albicans* was identified as the most prevalent species and account for 55.0% candidemia, followed by *Candida parapsilosis complex* (21.3%), *Candida tropicalis* (8.8%), *Candida glabrata complex* (7.5%), *Candida lusitaniae* (3.8%), and *Candida famata* (3.8%). The crude mortality at 30-days of candidemia was up to 30.0%, which is significantly higher than bacterial bloodstream infections (*p* = 0.006). Logistical analysis demonstrated that total parenteral nutrition >5 days (*p* = 0.036), urinary catheter >2 days (*p* = 0.001), distant organ metastasis of cancer (*p* = 0.002) and gastrointestinal cancer (*p* = 0.042) were the independent risk factors for candidemia.

**Conclusions:**

Candidemia showed significant higher mortality than bacterial bloodstream infections, *C. albicans* was cited as the primary pathogen. Total parenteral nutrition, urinary catheter, distant organ metastasis of cancer and gastrointestinal cancer are independent predictors for candidemia, this findings provides potential therapeutic targets for improving the outcome.

## Background

Candidemia is one of the most important nosocomial infections and associated with extremely high morbidity and mortality [1, 2]. It was cited as the fourth most prevalent nosocomial bloodstream infection in the United States and seventh to tenth in population-based studies, and the mortality is around 40% [1, 3–5]. A recent multi-center study, 183 US medical centers invovled, showed that candidemia ranked the first cause of primary bloodstream infections [3]. *Candida albicans* is the leading pathogens of candidemia worldwidely, but the shift in favor of non-*albicans Candida* species is occurring in recent years [1]. *Candida* species are the opportunistic pathogenic of human, and normally colonized in oral cavity, skin, and intestinal tracts in healthy individuals [1, 6]. They could trigger invasive infection in impaired immunological conditions, such as malignant diseases, immunodeficiency, and exposure to chemotherapy and antibiotics [1, 6, 7].

The poor prognosis of candidemia was proved be related with delayed of antifungal treatment for 12 h, which led to increased mortality up to three folds [8]. Though the rescue effect of echinocandin in case of delayed therapy, it has been reported the efficiency only among patients without severe underlying diseases [8–10]. It is a challenge to perform antifungal treatment promptly, since the hard to distinguish candidemia from bacterial bloodstream infections. The similar clinical symptoms are confusing, with shiver, fever and high ratio of neutrophils [11, 12]. The definitive diagnosis of candidemia mainly dependent on blood culture, which has low sensitive (38–50%) and time-consuming [13–15]. Therefore, risk factors model should be taken in consideration before establishing antifungal management strategies. Recent studies identified several risk factors for candidemia, including extreme age, prior antibiotics receiving, prior hemodialysis, parenteral nutrition, blood transfusion, abdomen as the portal of entry, and et al. [16, 17]. However, all risk factors mentioned above dramatically varied on population, living region, underlying diseases, and even the study period and methods applied [17–20].

Cancer patients are a large population with potential-risk to candidemia, both hematologic and solid malignancies were reported as the predictor for candidemia, especially for the former which were in high risk group compared to tumors in solid organs [18, 21, 22]. Meanwhile, candidemia could exacerbate the prognosis of malignant diseases (30-day mortality is up to 56%) [20, 23, 24]. Even though, the predictive risk models of candidemia among cancer patients are still limited.

As reported here, we performed a case-control study to analyze risk factors for candidemia among cancer patients during the period of 2006–2013. This may allow to apply antifungal prophylaxis to patients at greatest risk in time, and contribute to prognosis improvement [7].

## Methods

### Geography and setting

This study was performed at Tianjin Medical University Cancer Institute and Hospital (TMUCIH), among the top cancer institutes in China. TMUCIH possess a 2400 inpatient capacity, and serves both a population of 12,938,224 (at the end of 2010) of Tianjin, and also patients from other parts of China.

### Study design and data collection

This was a retrospective, case-control study to evaluate risk factors for cancer patients with candidemia. Subjects included cancer patients combined with hospital-acquired candidemia during 2006–2013 periods, and controls cancer patients diagnosed with nosocomial bloodstream infections of bacteria. Candidemia case was matched by two controls (gender, same inpatient period), case with recurrent bloodstream infections were excluded. Clinical data including age, gender, length of hospital stay, previous exposure to antibiotics, chemotherapy, neutropenia, surgery, types of cancer, distant organ metastasis of cancer, intensive care unit (ICU) admitted, indwelling devices, and crude mortality were collected. This study obtained the permissions from Bioethics Committee of Tianjin Medical University Cancer Institute and Hospital, and participants (consent to participate was obtained from all participants) to review patient records and use the data.

### Definitions

Types of cancer were differential diagnosed by pathological examination. Hospital-acquired bloodstream infection was defined as the first positive culture obtained at 48 h after hospital admission or 48 h of discharge, along with clinical signs of active infection. Candidemia and bacterial bloodstream infections were defined as the recovery of a pathogen in culture from blood at least once, except for the following species (at least twice), coagulase negative staphylococci, *Enterobacteriaceae* species, alpha-hemolytic streptococci, *Micrococcus* species, *Propionibacterium* species, *Corynebacterium* species, and *Bacillus* species. Recurrent bloodstream infection was defined as an episode of infection occurring at least 1 month after the initial diagnosis. Neutropenia was defined as an absolute neutrophil count of <1.5 × 10^9^/L. Cancer occurred in stomach, duodenum, colon and rectum were referred as gastrointestinal cancer. All the clinical data were collected within 30 days prior to the first positive blood culture, and crude mortality was referred to the ratio of death within 30 days after the first positive blood culture.

### Pathogens identification and antifungal susceptibility test

Blood samples (8–10 ml) were collected and auto-cultured by BACTEC 9050, 9120 or FX (Becton–Dickinson, Franklin Lakes, NJ, USA) for 5 days. Positive samples were sub-cultured on Sabouraud chloramphenicol agar or blood agar (JinZhangKeJi, Tianjin, China) at 35 °C for 24–48 h dependent on the results of gram staining. Species identification was performed on VITEK 2 Compact (bio-Merieux SA, Marcy l’Etoile, France). Antifungal susceptibility tests were performed using ATB FUNGUS 3 (bio-Merieux SA, Marcy l’Etoile, France).

### Statistical analysis

Statistical analyses were performed by SPSS 20.0 software (SPSS Inc., Chicago, IL, USA). The data of categorical variables are compared by Fisher’s exact test. Factors with a *p* value <0.05 in univariate tests were included in the logistic regression model for identifying independent risk factors. All tests were two-tailed, and significance level was set at *p* < 0.05.

## Results

### Distribution of pathogen species in cases and controls

A total number of 80 *Candida* species isolates were identified from 80 individuals of case groups. *C. albicans* was the predominant species (*n* = 44, 55.0%), followed by *C. parapsilosis* complex (*n* = 17, 21.3%), *C. tropicalis* (*n* = 7, 8.8%), *C. glabrata* complex (*n* = 6, 7.5%), *C. lusitaniae* (*n* = 3, 3.8%), and *C. famata* (*n* = 3, 3.8%). Table 1 showed the distribution of *C. albicans and* non-*albicans Candida* species in age, gender, ward and cancer type, and Fisher’s exact test indicated no significant difference.

**Table 1:** Distribution of *C. albicans* and non*-albicans Candida* in age, gender, ward and cancer type.

Profiles	*C. albicans*	non*-albicans Candida*	*p* value^a^
	(*n* = 44)	(*n* = 36)	
Age			0.655
> = 65 years old (%)	21(47.7%)	15(41.7%)	
< 65 years old (%)	23(52.3%)	21(58.3%)	
Gender			0.509
Male (%)	23(52.3%)	16(44.4%)	
Female (%)	21(47.7%)	20(55.6%)	
Ward			0.594
ICU (%)	8(18.2%)	10(27.8%)	
Surgery ward (%)	29(65.9%)	20(55.6%)	
Medicine ward (%)	7(15.9%)	6(16.7%)	
Cancer type			0.653
Gastrointestinal cancer (%)	24(54.5%)	17(47.2%)	
Non-gastrointestinal cancer (%)	20(45.5%)	19(52.8%)	

*ICU* intensive care unit

^a^Fisher’s exact test

In control group, 160 bacteria strains were isolated from 160 patients with bacterial bloodstream infections. Total number of 93 strains of gram negative bacteria were identified (58.1%). *Escherichia coli* was the major species (n = 35, 21.9%), followed by coagulase negative staphylococcus (*n* = 28, 17.5%), *Klebsiella pneumonia* (*n* = 19, 11.9%), enterococcus (*n* = 13, 8.1%), *Staphylococcus aureus* (*n* = 12, 7.5%), *Pseudomonas aeruginosa* (*n* = 9, 5.6%), enterobacter (*n* = 9, 5.5%) and others (*n* = 35, 21.9%).

### MICs of antifungal drugs for isolates of *Candidia* species

In vitro antifungal susceptibility (MIC range, MIC_50_ and MIC_90_) of *Candida* species isolates were presented in Table 2. Most isolates had low MICs to flucytosine, amphotericin B, fluconazole, itraconazole and voriconazole, and none was resistant to those antifungal drugs. However, several strains of *C. albicans, C. tropicalis* and *C. glabrata* complex showed higher MICs to fluconazole than others, i.e. 16 μg/ml for 1 strain of *C. albicans* and 1 *C. glabrata* complex, 8 μg/ml for 3 *C. glabrata* complex and 1 *C. tropicalis*, 4 μg/ml for 2 *C. glabrata* complex, 1 *C. albicans* and 2 *C. tropicalis*, respectively. Moreover, 2 strains of *C. albicans* had MICs of 16 μg/ml to flucytosine, with 1 *C. albicans* (0.25 μg/ml) and 3 *C. glabrata* complex (0.25 μg/ml for one and 0.5 μg/ml for the other two) exhibited higher MICs to itraconazole than others.

**Table 2:** In vitro antifungal susceptibility test results of *Candida* species

*Candida* species	Strains (n)	Antifungal agent	MIC Range (μg/ml)	MIC_50_ (μg/ml)	MIC_90_ (μg/ml)	No. (%) of susceptibility
*C.albicans*	44	Flucytosine	<=4–16	<=4	<=4	42(95.5%)
	44	Amphotericin B	<=0.5–1	<=0.5	1	ND
	44	Fluconazole	<=1–16	<=1	2	43(97.7%)
	44	Itraconazole	<=0.125–0.25	<=0.125	<=0.125	43(97.7%)
	44	Voriconazole	<=0.06–1	<=0.06	0.25	44(100%)
*C.parapsilosis* complex	17	Flucytosine	<=4	<=4	<=4	17(100%)
	17	Amphotericin B	<=0.5–1	<=0.5	<=0.5	ND
	17	Fluconazole	<=1–2	1	1	17(100%)
	17	Itraconazole	<=0.125	<=0.125	<=0.125	17(100%)
	17	Voriconazole	<=0.06–0.5	<=0.06	0.5	17(100%)
*C.tropicalis*	7	Flucytosine	<=4	<=4	<=4	7(100%)
	7	Amphotericin B	<=0.5	<=0.5	<=0.5	ND
	7	Fluconazole	<=1–8	<=1	4	7(100%)
	7	Itraconazole	<=0.125	<=0.125	<=0.125	7(100%)
	7	Voriconazole	<=0.06–0.5	0.12	0.5	7(100%)
*C.glabrata complex*	6	Flucytosine	<=4	<=4	<=4	6(100%)
	6	Amphotericin B	<=0.5	<=0.5	<=0.5	ND
	6	Fluconazole	4–8	8	16	5(83.3%)
	6	Itraconazole	0.125–0.5	0.25	0.5	3(50%)
	6	Voriconazole	<=0.06–0.5	0.25	0.5	6(100%)
*C.lusitaniae*	3	Flucytosine	<=4	<=4	<=4	3(100%)
	3	Amphotericin B	<=0.5	<=0.5	<=0.5	ND
	3	Fluconazole	<=1	<=1	<=1	3(100%)
	3	Itraconazole	<=0.125	<=0.125	<=0.125	3(100%)
	3	Voriconazole	<=0.06	<=0.06	<=0.06	3(100%)
*C.famata*	3	Flucytosine	<=4	<=4	<=4	3(100%)
	3	Amphotericin B	<=0.5	<=0.5	<=0.5	ND
	3	Fluconazole	<=1	<=1	<=1	3(100%)
	3	Itraconazole	<=0.125	<=0.125	<=0.125	3(100%)
	3	Voriconazole	<=0.06–0.12	<=0.06	0.12	3(100%)

*NA* Not Defined

### Clinical characteristics of patients in cases and controls

During the period of 2006–2013, total number of 80 enrolled cancer patients experienced one episode candidemia, and 160 controls cancer patients with one episode bacterial bloodstream infections according to the criterions. The gender frequency was equal for cases (39 male patients, 48.8%) and controls (78 male patients, 48.8%). The median age for cases was 64 years old (range from 33 to 93 years old), and 62 years old for controls (range from 19 to 90 years old). Cancer types for cases and controls were listed below, 41 (51.2%) versus 30 (18.8%) for gastrointestinal cancer, 9 (11.3%) versus 28 (17.5%) for pancreatic cancer, 7 (8.8%) versus 24 (15.0%) for lung cancer, 6 (7.5%) versus 18 (11.3%) for hepatic cancer, 6 (7.5%) versus 20 (12.5%) for bile duct cancer, 5 (6.3%) versus 11 (6.9%) for ovarian cancer, 3 (3.8%) versus 12 (7.5%) for cervix cancer, 2 (2.5%) versus 5 (3.1%) for breast cancer, and 1 (1.3%) versus 12 (7.5%) for kidney cancer.

### Risk factors for candidemia

Table 3 showed the clinical characteristics for cases and controls. Ratio of patients with length of hospital stay > = 30 days, Mechanical ventilation >2 days and ICU admitted >3 days were slightly higher in cases (31.3%, 10.0% and 22.5%, respectively) than controls (22.5%, 4.4% and 15.6%, respectively), but these differences were not significantly by Fisher’s exact test (*p* = 0.158, 0.098 and 0.213, respectively).

**Table 3:** Clinical characteristics for cases and controls

Clinical characteristics	Cases	Controls	*p* value^a^
	(80 cases)	(160 cases)	
Male (%)	39 (48.8)	78 (48.8)	1.000
Age > = 65 years old (%)	36 (45.0)	40 (25.0)	0.002
Length of hospital stay > = 30 days (%)	25 (31.3)	36 (22.5)	0.158
Surgery (%)	57 (71.2)	83 (51.9)	0.005
Central venous catheters >7 days (%)	63 (78.8)	95 (59.4)	0.003
Total parenteral nutrition >5 days (%)	48 (60.0)	23 (18.1)	<0.001
Urinary catheter >2 days (%)	61 (76.2)	59 (36.9)	<0.001
Nasogastric tube >3 days (%)	44 (55.0)	39 (24.4)	<0.001
Mechanical ventilation >2 days (%)	8 (10.0)	7 (4.4)	0.098
Neutropenia (%)	11 (13.8)	24 (15.0)	0.849
Chemotherapy (%)	46 (57.5)	100 (62.5)	0.271
Distant organ metastasis of cancer (%)	32 (40.0)	27 (16.9)	<0.001
Gastrointestinal cancer (%)	41 (51.3)	30 (18.8)	<0.001
ICU admitted >3 days (%)	18 (22.5)	25 (15.6)	0.213
Medicine ward (%)	13 (16.2)	50 (31.2)	0.013
Surgery ward (%)	49 (61.3)	85 (53.1)	0.271
Previous antibiotics exposure (%)	75 (93.8)	130 (81.3)	0.011

*ICU* intensive care unit

^a^Fisher’s exact test

By Fisher’s exact test, age > = 65 years old (*p* = 0.002), surgery (*p* = 0.005), central venous catheters >7 days (*p* = 0.003), total parenteral nutrition >5 days (*p* < 0.001), urinary catheter >2 days (*p* < 0.001), nasogastric tube >3 days (*p* < 0.001), distant organ metastasis of cancer (*p* < 0.001), gastrointestinal cancer (*p* < 0.001) and previous antibiotics exposure (*p* = 0.011) were associated with candidemia. Medicine ward admitted seemed as a predictor for candidemia (16.2% cases versus 31.2% controls, *p* = 0.013).

Logistical analysis indicated that total parenteral nutrition >5 days (OR = 2.515, 95% CI = 1.060–5.966, *p* = 0.036), urinary catheter >2 days (OR = 5.105, 95% CI = 1.913–13.626, *p* = 0.001), distant organ metastasis of cancer (OR = 3.610, 95% CI = 1.578–8.263, *p* = 0.002) and gastrointestinal cancer (OR = 2.189, 95% CI = 1.029–4.657, *p* = 0.042) were the independent risk factors for candidemia, Table 4.

**Table 4:** Risk factors for candidemia among cancer patients

Risk factors	OR	95% CI	*p* value
Age > = 65 years old	0.989	0.502–1.947	0.974
Surgery	1.097	0.348–3.455	0.874
Central venous catheters >7 days	1.64	0.729–3.689	0.232
Total parenteral nutrition >5 days	2.515	1.060–5.966	0.036
Urinary catheter >2 days	5.105	1.913–13.626	0.001
Nasogastric tube >3 days	1.024	0.423–2.480	0.958
Distant organ metastasis of cancer	3.610	1.578–8.263	0.002
Gastrointestinal cancer	2.189	1.029–4.657	0.042
Medicine ward	1.107	0.435–2.816	0.830
Previous antibiotics explore	1.242	0.401–3.847	0.707

*OR* odds ratio, *CI* confidence interval

### Crude mortality of candidemia

In this study, 24 candidemia patients deceased within 1 month after recovery of an isolate leading to a crude mortality of 30.0%, which was significantly higher than controls (*n* = 23, 14.4%, *p* = 0.006). The crude mortality varied among *Candida* species but without significant difference (*p* = 0.382). *C. tropicalis* had the highest mortality (*n* = 4, 57.1%), and followed by *C. glabrata* complex (*n* = 3, 50.0%), *C. lusitaniae* (*n* = 1, 33.3%), *C. albicans* (*n* = 12, 27.3%) and *C. parapsilosis* complex (*n* = 4, 23.5%). None of patient with candidemia due to *C. famata* was dead within 1 month during the whole study period.

## Discussion

Compared with other underlying diseases, cancer patients are much more susceptible to candidemia and show higher mortality [23, 25]. In this study, the crude mortality for cancer patients with candidemia was reported up to 30.0%, about two folds higher than patients with bacterial bloodstream infections. Prophylactic and empirical antifungal therapies are highly suggested in patients with malignant disorders [19, 25, 26]. However, it is hard to determine time point to prophylactic antifungal treatment, since the potential risk factors in each individuals are hard to identify. In addition, improper prophylactic treatments select in favor of non-*albicans* Candida species (several species have high MICs to azoles) infections, and the severe hepatorenal toxicity of antifungal drugs would worse the underlying diseases [27, 28]. In the present study, we provided the candidemia etiology and performed a case-control study to evaluate risk factors of candidemia among cancer patients, which would contribute to the strategies of antifungal therapy and prognosis improvement.

We reported here that, susceptibility test of antifungal drugs were performed for all isolates of *Candida* species. Concern need be addressed on those isolates of *Candida* species (including *C. albicans, C. tropiticals* and *C. glabrata* complex) which had higher MICs to fluconazole than others, for the increasing MICs to fluconazole and liner linkage of MICs between fluconazole and voriconazole among *Candida* species as reported [29]. We validated that isolates of *C. lusitaniae* and *C. famata* were susceptible to the antifungal drugs. However, the antifungal susceptibility profile of rare species is poorly studied worldwide, and the current MICs interpretive may not be suitable for some rare species. For example, amphotericin B has been reported showing little effect on treatment of *C. lusitaniae* fungemia, even though it was proved originally susceptible to amphotericin B in vitro [30, 31].

Total parenteral nutrition and urinary catheter have been suggested as candidemia predictors by other researchers, which is consistent with our findings. Meanwhile, we reported here that distant organ metastasis of cancer and gastrointestinal cancer were proved as independent predictors for candidemia, which have been rarely reported by others [15–17, 32]. Distant organ metastasis of cancer is considered as the late stage of cancer, and patients are usually treated as impaired immune system and submitted to aggressive therapy, which may let to susceptible to candidemia. Gastrointestinal system, especially the upper gastrointestinal track, is recognized as the habitat of *Candida* species [33, 34]. It is reported that the incidence of abdominal candidiasis was 41.0% in gastroduodenal perforations, whereas 11.8% in colorectal perforations [33]. *Candida* species may translocate into bloodstream from impaired mucosa barrier of gastrointestinal in cancer, led to the infection [35]. Previous studies concluded that the lesions in gastrointestinal mucosa caused by invasive procedures such as nasogastric tubes imbedding and gastric acid suppressants, were the independent predictors for candidemia [5, 36]. However, more clinical data and further studies need be collected to both distant organ metastasis of cancer and gastrointestinal cancer are the risk factors to the susceptibility to candidemia for cancer patients.

Among enrolled patients in our study, age > = 65 years old, indwelling central venous catheters, surgery and previous antibiotics exposure were related with candidemia, but not considered as independent predictors, since these factors were not tightly consistent in previous reports [7, 17, 18, 20, 32]. The inconsistent findings may vary on the population, region and study design. For instance, central venous catheters has been excluded as independent indicator for candidemia when studies were designed to compare candidemia from different species of *Candida* [33, 37, 38]. We speculated that combined risk factors (cumulative number) might predict candidemia more accurately than single factor, which need further validations.

Neutrophils are considered as the first barrier in anti-infections reactions, and neutropenia is one of the most important indicators for impaired immune system [16]. However, the association of neutropenia with candidemia is still conflict. Karabinis A et al. proved that neutropenia significantly increase the risk of candidemia in cancer patients [20]. A study in Taiwan suggested that neutropenia predict the infection of non-*albicans Candida* species [39]. Another study preformed in Mainland of China found that candidiasis are more likely to occur in non-neutropenia patients in ICU [38]. We also did not prove the relationship between neutropenia and candidemia in this study. In recent years, drugs which can elevate the total number of neutrophils are widely prescribed among patients receiving chemotherapy or other immunosuppressive therapeutics, which may contribute to underestimated ratio of neutropenia patients, as well as the contribution of neutropenia to candidemia.

In addition, several risk factors recognized in other studies have been exhibited little association with candidemia in our report, including length of hospital stay > = 30 days, mechanical ventilation, chemotherapy and ICU admitted. Excepted the number of mechanical ventilation and ICU admitted were too low to without bias, the remaining could be real no discrepancies between cases and controls, since both cases and controls were cancer patients and most of them received long inpatient stay and chemotherapy [40, 41].

The crude mortality of candidemia are slightly differences when it comes to species and not consistent in different studies. In our study, *C. glabrata* complex and *C. tropicalis* are linked to higher crude mortality, consistent to previous studies (the mortality of *C. glabrata* complex and *C. tropicalis* ranged from 44.7% to 61%) [36, 42, 43]. Meanwhile, *C. parapsilosis* complex exhibited low mortality in both our and other reports, which was considered to be associated with the less virulent of them [43, 44]. However, the crude mortality caused by *C. albicans* exhibited a wide variation, slightly higher than *C. parapsilosis complex* in our study whereas ranked the highest mortality in other report [45]. Different study period and underlying diseases might contribute to the conflicted conclusions.

## Conclusions

In conclusion, candidemia exhibited higher mortality than bacterial bloodstream infections among cancer patients. *C. albicans* contributed as the leading pathogen for candidemia. Total parenteral nutrition >5 days, urinary catheter >2 days, distant organ metastasis of cancer and gastrointestinal cancer are the independent risk factors for candidemia, which allow us to define patients at potential risk and perform prophylaxis therapies in short time. There are, however several limitations in the present study need be further studied. In this retrospective study, *Candida* index was not included, and causations of low number of patients with neutropenia were not been confirmed. Furthermore, all patients enrolled in a single center, the size and diversity of samples are limited. In future, prospective multi-center study with large sample size should be performed, which could provide more relevant epidemiology information.

## Abbreviations

*C.*: *Candida*
ICU: Intensive care unit
TMUCIH: Tianjin Medical University Cancer Institute and Hospital
